# An Effective Somatic-Cell Regeneration and Genetic Transformation Method Mediated by *Agrobacterium tumefaciens* for *Portulaca oleracea* L.

**DOI:** 10.3390/plants13172390

**Published:** 2024-08-27

**Authors:** Mengyun Xu, Xinyu Zhao, Jiahui Fang, Qinwen Yang, Ping Li, Jian Yan

**Affiliations:** Key Laboratory of Agro-Environment in the Tropics, College of Natural Resources and Environment, South China Agricultural University, Guangzhou 510642, China; xumengyun1018@stu.scau.edu.cn (M.X.); 15806458017@163.com (X.Z.); fangjiahui1109@gmail.com (J.F.); yangcreativity@foxmail.com (Q.Y.); liping2016@scau.edu.cn (P.L.)

**Keywords:** *Portulaca oleracea* L., genetic transformation, *Agrobacterium tumefaciens*, tissue culture

## Abstract

Purslane (*Portulaca oleracea* L.) is highly valued for its nutritional, medicinal, and ecological significance. Genetic transformation in plants provides a powerful tool for gene manipulation, allowing for the investigation of important phenotypes and agronomic traits at the genetic level. To develop an effective genetic transformation method for purslane, various organ tissues were used as explants for callus induction and shoot regeneration. Leaf tissue exhibited the highest dedifferentiation and regeneration ability, making it the optimal explant for tissue culture. By culturing on Murashige and Skoog (MS) medium supplemented with varying concentrations of 6-benzyleaminopurine (6-BA) and 1-naphthaleneacetic acid (NAA), somatic cells from leaf explants could be developed into calli, shoots, and roots. The shoot induction results of 27 different purslane accessions elucidated the impact of genotype on somatic-cell regeneration capacity and further confirmed the effectiveness of the culture medium in promoting shoot regeneration. On this basis, a total of 17 transgenic plants were obtained utilizing the genetic transformation method mediated by *Agrobacterium*. The assessment of GUS staining, hygromycin selection, and polymerase chain reaction (PCR) amplification of the transgenic plants as well as their progeny lines indicated that the method established could effectively introduce foreign DNA into the purslane nucleus genome, and that integration was found to be stably inherited by offspring plants. Overall, the present study demonstrates the feasibility and reliability of the *Agrobacterium*-mediated genetic transformation method for introducing and integrating foreign DNA into the purslane genome, paving the way for further research and applications in purslane genetic modification.

## 1. Introduction

*Portulaca oleracea* L., commonly known as purslane, is an annual succulent herb belonging to the Portulacaceae family [1]. With a rich nutrient content and active chemical ingredients, purslane has been valued for centuries both as a vegetable and folk medicine [2]. Purslane serves as an excellent source of ω-3 fatty acids, along with vitamins A, C, and E, as well as essential minerals, thus making it a valuable addition to a balanced diet [3,4]. Due to its anti-inflammatory, antioxidant, and antimicrobial properties, purslane is widely utilized in various traditional medicine systems [5,6]. Moreover, purslane holds the distinction of being the eighth most cosmopolitan weed due to its remarkable tolerance to abiotic stress [7,8]. Particularly noteworthy is its unique adaptation strategy in response to drought stress, make it a valuable model system for studying integrated C_4_/CAM photosynthetic metabolism mechanisms [9,10].

As an important plant used for both medicinal and culinary purposes, purslane has recently attracted increasing attention. With the completion of a high-quality chromosome-level genome assembly, there is a growing emphasis on understanding the exploration of the molecular mechanisms underlying its phenotypic traits and the utilization of related genetic resources [11]. Plant genetic transformation, involving the introduction of foreign DNA into an organism’s genome, provides a powerful tool for gene manipulation, editing, and validation [12,13]. This technique plays a pivotal role not only in deciphering gene function but also in advancing molecular breeding strategies. It is particularly significant for the genetic enhancement of target traits in crops, as it can streamline the breeding process and substantially shorten the duration of breeding cycles [14]. Plant genetic transformation has undergone several decades of development since the creation of transgenic tobacco plants, resulting in the establishment of various methods such as *Agrobacterium*-mediated transformation, the floral dip method, pollen tube pathway-mediated genetic transformation, the gene gun method, and the chemical infiltration method [12,15,16,17]. Among these, *Agrobacterium*-mediated transformation is the most widely utilized approach. It is favored for its ability to introduce a single copy of the transgene into the host genome with relatively high efficiency, leading to stable and predictable gene expression levels [18].

*Agrobacterium* demonstrates the capability to introduce foreign DNA fragments or gene editors carried by Ti plasmids, Ri plasmids, and other carriers into host cells, thereby integrating them into the host genome [19]. This process enables the stable expression of the target gene in the host plant, which can be inherited by subsequent generations. Until now, genetic transformation methods mediated by *Agrobacterium* have been established for a significant number of plant species, including tobacco, rice, maize, tomato, and soybean [12,20,21,22]. For species in the Portulaceae family, the transformation of *Portulaca grandiflora* was performed with *Agrobacterium tumefaciens* strains A281 and T1272; Sedaghati et al. developed a method for Iranian purslane using sonication and vacuum infiltration with somatic embryogenesis [23,24]. Genetic transformation is largely dependent on the plant regeneration ability of somatic cells. Various factors, including the genotype of plant species, the type of explants used from different organ tissues, and the developmental stage, play a significant role in determining the regeneration ability and transformation efficiency. Thus, understanding these variables is crucial for optimizing the genetic transformation process for different plant genotypes [25,26].

The aim of this study is to develop an effective somatic-cell regeneration and *Agrobacterium*-mediated genetic transformation system for purslane. Wild common purslane was used as the recipient plant, and various plant tissues, including the leaf, stem, root, and seed, were utilized as explants for callus induction to determine the best organ for tissue culture. Ultimately, we established a system for the somatic-cell dedifferentiation and redifferentiation of purslane through the optimization of callus induction and shoot regeneration media. We also successfully developed a genetic transformation method mediated by *Agrobacterium*, enabling the introduction of foreign genes into the genome of purslane. The development of this genetic transformation system will provide essential technical tools for the functional analysis of purslane genes and the exploration of molecular mechanisms underlying its key traits.

## 2. Results

### 2.1. Callus Induction of Different Parts of Purslane and Medium Optimization

**Figure 1:**
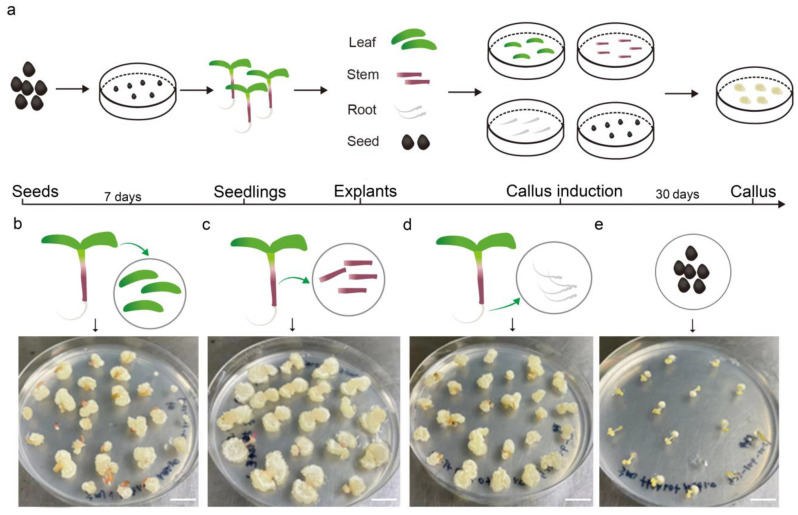
Callus induction using the leaves, stems, roots, and seeds from aseptic seedlings of *Portulaca oleracea* L. (**a**) The workflow of explant treatment and callus induction. (**b**–**e**) Callus induction using the leaf, stem, root, and seed as explants, respectively. Photos of the callus after one month of induction. Bar is 1 cm.

To determine the optimal organ for tissue culture in purslane, different parts, including the leaf, stem, root, and seed, were used for callus induction in the medium (MS, 30 g/L sucrose, 7 g/L agar, 1 mg/L NAA, and 1 mg/L 6-BA). After one month of cultivation, the callus induction rates were statistically analyzed. The results indicated that the leaf and root tissues exhibited a high callus induction rate, nearly reaching 100%, whereas the seed exhibited a relatively low induction rate (Figure 1). Additionally, the resulting callus tissue from the seed exhibited a hard texture and slow growth, with its surface characterized by fine hairs, which may pose challenges for subsequent redifferentiation processes. On the other hand, the callus derived from the stem exhibited relatively rapid growth but had a higher water content. This trait made it prone to vitrification and browning upon subculture, which in turn was unfavorable for shoot regeneration and redifferentiation. In contrast, leaf explants showed a relatively high efficiency in callus induction, generating a dense and smooth-textured callus with rapid growth upon subculture. Given its ease of acquisition, manipulability, and sterilization, the leaf was considered the most suitable organ for callus induction and tissue culture.

To optimize the appropriate concentration of hormone for callus induction, leaf explants were placed on media with various hormone concentrations. The callus induced and cultured under conditions of low-hormone-concentration medium exhibited a semi-transparent appearance, elevated water content, and fine trichomes on an irregular surface texture (Figure S1). Conversely, the callus cultivated in a high-hormone-concentration medium exhibited a whitish hue and hard texture. Given the subsequent necessity for callus redifferentiation into shoots and roots, the use of high concentrations of cytokinin is not recommended. Therefore, MS medium supplemented with 2 mg/L 6-BA and 1 mg/L NAA was considered the appropriate callus induction and subculture medium for purslane leaf explant. On this medium, the callus induction rate could reach up to 100%, and the induced calli were able to synthesize chlorophyll and undergo greening after transfer to light conditions. Additionally, they could develop into shoots when transferred to shoot regeneration medium for more than one month (Figure S1).

### 2.2. Shoot Regeneration and Medium Optimization

**Figure 2:**
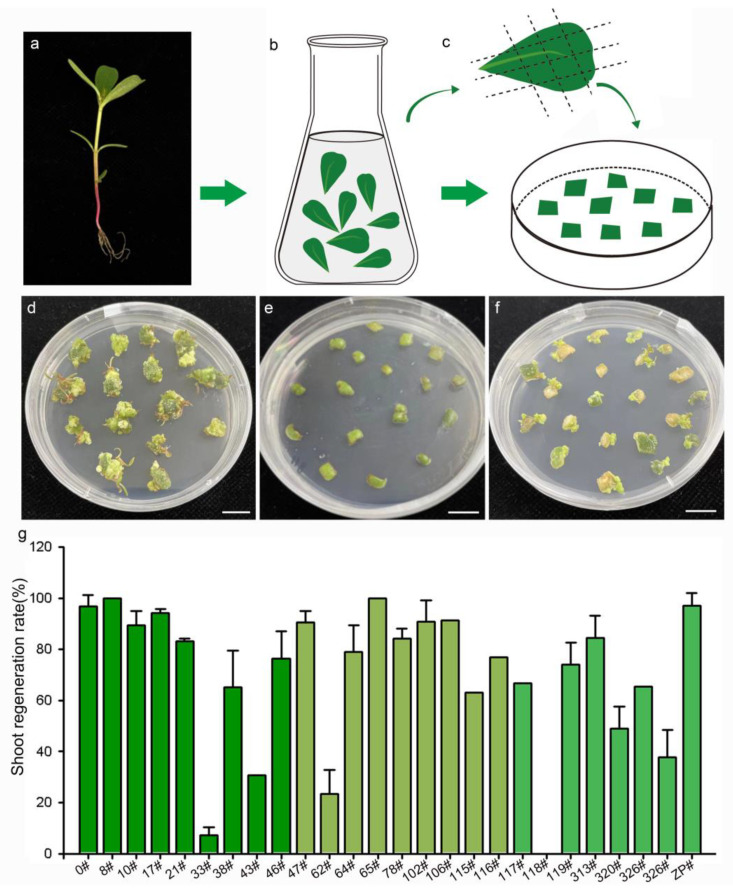
Shoot regeneration of 27 purslane accessions representing different genotypes. (**a**) Soil-planted purslane seedings. (**b**) Leaf sterilization. (**c**) Leaf explants cultured on the shoot regeneration medium. (**d**–**f**) Photos of purslane accession 0#, 118#, and 47# after 50 days of induction, respectively. (**g**) Statistics of the shoot regeneration ratio of purslane leaves from 27 accessions. Values are means ± SD (n = 3). Bar is 1 cm.

Somatic-cell redifferentiation and shoot regeneration are key steps in the plant genetic transformation system. To optimize the medium for shoot regeneration, leaf explants were cultured on media with various concentrations of hormone (Table S1). The response of the explants showed that the MS medium supplemented with 2 mg/L 6-BA was the appropriate medium. On this medium, somatic cells could regenerate into shoots and rootless plantlets after cultivation for 50 days (Figure 2). The regenerated plantlets were capable of developing roots on the rooting medium and survived to exhibit normal growth after being transplanted into soil. In addition to leaf explants, stem and root segments were also cultured on this medium and exhibited the ability to develop into shoots with a relatively low regeneration rate (Figure S2).

Previous studies have demonstrated that the genotype of plant material is a significant factor influencing the processes of somatic-cell dedifferentiation and redifferentiation [27]. To evaluate the effectiveness of the medium for shoot regeneration and the impact of genotype on the regeneration response, 27 purslane accessions from various locations in China were cultured on MS medium supplemented with 2 mg/L 6-BA. The origin information of the 27 different purslane accessions is listed in Table 1. The results indicated that purslane accessions with different genotypes exhibited various responses to induction and culture (Figure 2). Explants from purslane accession 0#, originating from Guangzhou, China, exhibited rapid growth on the medium and subsequently redifferentiated into shoots (Figure 2d). In contrast, accession 118# nearly ceased growth and failed to regenerate shoots (Figure 2e). Accession 47# exhibited relatively slower growth but was able to develop shoots, with a high induction rate of 90% (Figure 2f,g). Figure 2g presents the statistical results for the shoot induction rates of the 27 different purslane accessions. Among them, 22 accessions exhibited shoot redifferentiation rates exceeding 60%. Therefore, the shoot regeneration medium, MS medium supplemented with 2 mg/L 6-BA, was demonstrated to be effective for shoot induction and differentiation in the majority of purslane accessions, particularly for accession 0#, which was used as the recipient plant material in this study for *Agrobacterium*-mediated genetic transformation.

### 2.3. The Developmental Directions of Leaf Explants Vary on Different Media

**Figure 3:**
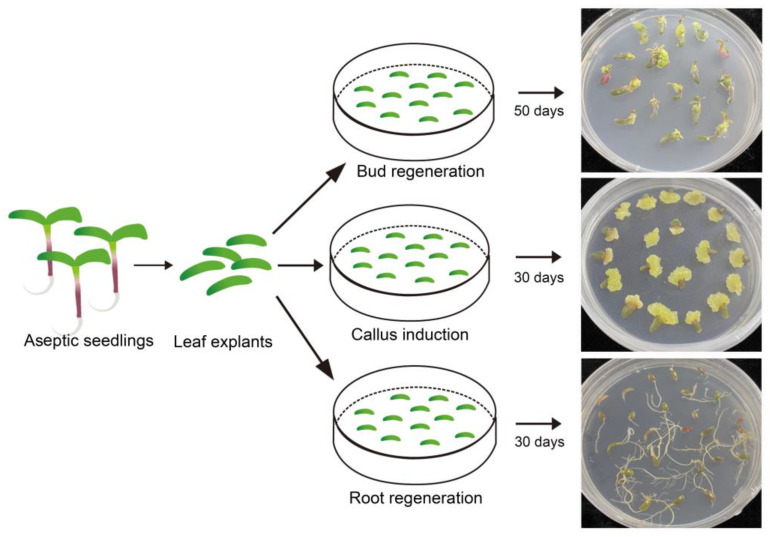
Results of leaf explants from sterilized *Portulaca oleracea* L. seedlings induced by different types of media. Bar is 1 cm.

To further validate the effectiveness of the induction media used in this study, leaf explants from sterile seedlings were inoculated on different types media for callus induction as well as shoot and root regeneration. The responses of leaf explants after one month of treatment indicated that same somatic cells from leaf tissue could respectively develop into calli, shoots, and roots under the influence of 6-BA, NAA, and their combination (Figure 3). This finding, that identical somatic cells exhibited the capability to grow and develop in completely distinct directions, further validated the effectiveness and stability of the callus induction, shoot regeneration, and rooting media.

### 2.4. Sensitivity of Seedlings and Explants to Antibiotics

Antibiotic selection is an important procedure in *Agrobacterium*-mediated genetic transformation systems [28]. The selection pressure exerted by appropriate concentrations of antibiotics can effectively screen transgenic plants without adversely affecting their normal growth by inhibiting the growth of non-transgenic cells [29,30,31]. Different plant species exhibit significant variability in their sensitivity to antibiotics. Low selection pressure may result in false-positive plants, while excessive concentrations of antibiotics would adversely affect the normal growth and survival of transgenic plants. Therefore, testing the responses of purslane to different concentrations of antibiotics and determining the appropriate selection pressure is a key step in constructing an *Agrobacterium*-mediated transgenic system. Currently, the antibiotic marker genes carried by common binary vectors include kanamycin, hygromycin, and basta [29,30,31]. In the present study, the sensitivity of purslane seed and leaf explants to kanamycin and hygromycin was assessed to establish a reference for the subsequent screening of transgenic plants.

**Figure 4:**
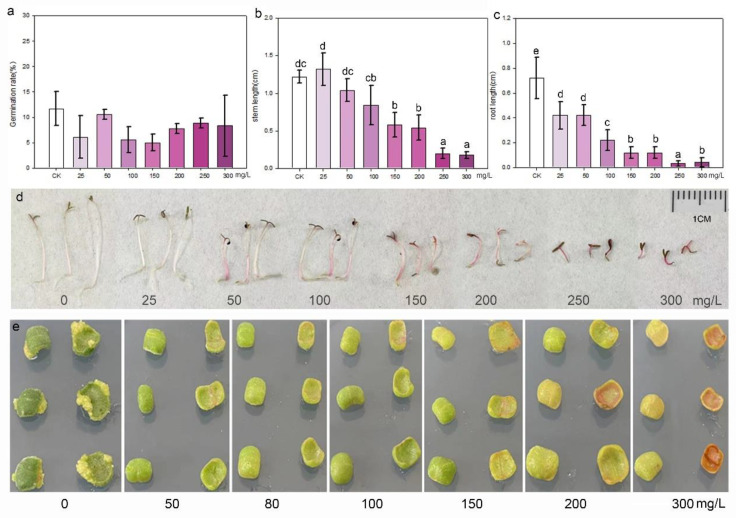
Kanamycin sensitivity identification of seedlings and leaf explants of purslane. (**a**) Germination rate; values are means ± SD (n = 3). (**b**) Stem length; values are means ± SD (n = 5). (**c**) Root length; values are means ± SD (n = 5). (**d**) Growth of purslane seedlings in aqueous solution with various kanamycin concentrations. (**e**) Shoot induction of leaf explants in media with various kanamycin concentrations. The data in (**a**–**c**) were analyzed by one-way ANOVA multiple comparisons followed by LSD tests. Different letters above the bars indicate a significance at *p* < 0.05.

Purslane seeds were cultured in aqueous solutions containing different concentrations of kanamycin to assess their sensitivity to the antibiotic during germination and growth. The statistical result indicated that kanamycin did not affect the germination of purslane seeds. There was no significant difference in the germination rate between the control and treatment groups, even at concentrations as high as 300 mg/L (Figure 4a). However, kanamycin significantly inhibited the growth of purslane seedlings, particularly affecting root elongation (Figure 4b–d). Even at a low concentration of 25 mg/L, root growth was stunted, resulting in a significant difference compared to the control. Purslane leaf explants were inoculated on shoot induction media with different concentrations of kanamycin, which revealed that kanamycin severely inhibited the growth and redifferentiation of explants (Figure 4e). After explants were cultured on the shoot regeneration medium for 30 days, some white shoot primordium tissues appeared at the margin of the explants in contact with the medium. However, this phenomenon was not observed in the groups treated with kanamycin. Moreover, as the concentration of kanamycin increased, the explants began to undergo browning and subsequently died. Based on these results, it is recommended to use a kanamycin concentration of 50 mg/L for the selection and screening of transgenic cells and plants.

The same experiments were conducted for purslane to identify its sensitivity to hygromycin. The results showed that purslane seeds and explants were highly sensitive to hygromycin, with even a low concentration of 5 mg/L causing the significant inhibition of growth in purslane leaf explants. Compared to the control, a hygromycin concentration of 25 mg/L significantly inhibited both the elongation of purslane seedling hypocotyls and root growth (Figure S3). Despite the efficacy of low concentrations of hygromycin in selecting positive transgenic plants, the antibiotic’s impact may detrimentally affect subsequent rooting and plant survival. Consequently, for purslane, hygromycin is not recommended as the marker for screening transgenic plants.

### 2.5. Genetic Transformation Workflow Mediated by Agrobacterium tumefaciens

**Figure 5:**
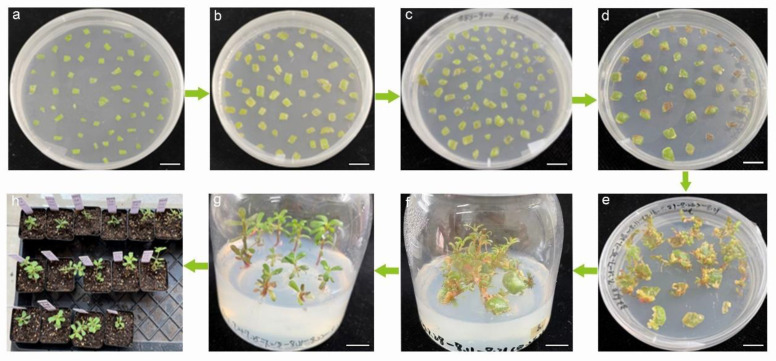
Workflow of genetic transformation of using leaf explants from soil-planted seedlings of *Portulaca oleracea* L. mediated by *Agrobacterium tumefaciens.* (**a**) Preculture. (**b**) *Agrobacterium* co-culture. (**c**) Bacteriostasis. (**d**–**f**) Shoot regeneration. (**g**) Root regeneration. (**h**) Transplant. Bar is 1 cm.

Based on the systematic identification of purslane somatic-cell dedifferentiation and redifferentiation, including callus induction and shoot and root regeneration, *Agrobacterium* strain GV3101 with the pCAMBIA1301-35SN plasmid was employed to infect purslane leaf explants. Our aim was to introduce foreign genes into the purslane genome, thereby establishing an effective and stable genetic transformation system. The main procedures in the workflow included preculture, *Agrobacterium* infection, co-cultivation, antibacterial treatment, shoot regeneration, rooting, and transplanting seedlings (Figure 5). Preculture was the first procedure that placed the sterilized leaf explants on the medium with a lower concentration of 6-benzylaminopurine to acclimate the wounded explants to in vitro culture and increase the survival rate of somatic cells after *Agrobacterium* infection. Previous studies have shown that mitotically active cells in the division phase are more easily infected and transformed by *Agrobacterium*. Therefore, culturing explants to an optimal state can increase the number of mitotically active cells and improve the rate of genetic transformation [32]. The optimal preculture time of explants was suggested as 1–4 days [32,33]. In this study, 2 days of preculture for the leaf explants were employed. For co-cultivation, leaf explants infected with *Agrobacterium tumefaciens* were transferred to the shoot regeneration medium with 100 mmol/L acetosyringone at 28 °C in dark conditions for 2 days. Virulence (*vir*) genes are located on the Ti plasmid and encode proteins that are essential for the transfer of T-DNA from the bacterium into the plant host cell [12]. Acetosyringone functions as a *vir* gene inducer in *Agrobacterium*-mediated genetic transformation. It enhances the expression of *vir* genes, which are essential for the transfer of T-DNA from the bacterium to the plant cells [12].

After infection and co-cultivation, 200 mg/L timentin was added to the medium to inhibit the overgrowth of *Agrobacterium*. The clean explants without obvious *Agrobacterium* on their surfaces were transferred to shoot regeneration medium containing timentin. The shoots showing relatively healthy growth were transferred to rooting medium. To increase survival rate, the regenerated plantlets with strong and healthy roots were removed from the rooting medium and kept in conical flasks with sterilized water for 3 days. Subsequently, they were transferred to soil for normal growth. Further identification was performed to verify the transgenic plants.

### 2.6. Transgenic Plant Identification

**Figure 6:**
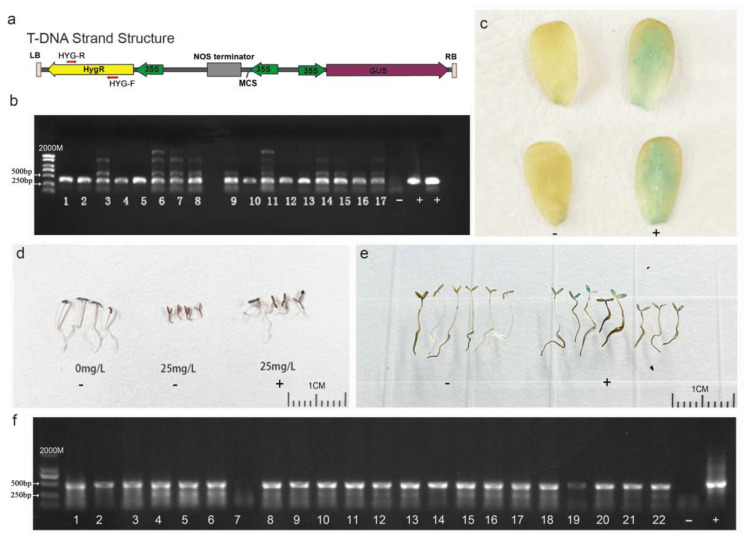
Identification of transgenic purslane plants and their progenies. (**a**) T-DNA sequence feature of pCAMBIA1301-35SN. (**b**) PCR identification of T_0_ transgenic plants with specific hygromycin primers. (**c**) GUS staining of T_0_ transgenic plants. (**d**) Hygromycin solution screening of T_1_ transgenic plants. (**e**) GUS staining of transgenic plant progenies. (**f**) PCR Identification by specific hygromycin primers for T_1_ transgenic plant progenies. ‘-’ represents wild type, ‘+’ represents the plasmid-positive control (**b**,**f**) and positive transgenic plants (**c**,**d**,**e**). Bar is 1 cm.

To identify whether the foreign plasmid DNA sequence was integrated into the genome of purslane plants that regenerated from the leaf explants and underwent *Agrobacterium* infection, co-cultivation, and somatic-cell regeneration, total DNA of the purslane plants was extracted, and specific primers, as illustrated in Figure 6a, were designed to amplify the hygromycin gene carried by the vector. The result shown in Figure 6b indicated that a total of 17 positive transgenic plants were identified from more than 100 regenerated plants. The GUS reporter gene, carried by the pCAMBIA1301-35SN plasmid, encodes a β-glucuronidase protein capable of hydrolyzing X-Gluc into a blue compound. This reaction results in blue coloration or blue spots in the plant tissues [34]. Therefore, GUS staining can be used to detect the expression of the foreign gene in transgenic plants at the protein level. In the present study, the young leaves of the purslane plants were collected for GUS staining to further confirm the integration of the foreign gene. Compared to those of the non-transgenic plants, the leaves of positive transgenic plants exhibited visible blue staining with X-Gluc (Figure 6c). According to the results above, the foreign gene was successfully integrated into the purslane genome by *Agrobacterium*-mediated genetic transformation, and it could be stably expressed and translated into active protein in the purslane’s somatic cells.

To determine whether the foreign DNA fragments introduced to the purslane genome can be stably transmitted to the progeny lines, T_1_ generation seeds harvested from the T_0_ transgenic plants were germinated in a water solution with 25 mg/L hygromycin. PCR amplification and GUS staining were also conducted for further identification. The results are shown in Figure 6d–f. In contrast to non-transgenic plants, the growth of which was severely inhibited by hygromycin, transgenic plants with the DNA fragment of the plasmid exhibited relatively normal growth. Although the growth of transgenic plants was somewhat affected by hygromycin, the plants were still able to develop roots and expand cotyledons (Figure 6d). The GUS staining of progeny seedlings showed that the cotyledons of the positive plants were colored a visible blue (Figure 6e), but not all the seedlings exhibited the blue color. Combining the PCR amplification result that not all the samples showed the positive band (Figure 6f), it can be inferred that the foreign DNA fragment was segregated in the offspring lines. This indicated that the T-DNA fragment of the plasmid was integrated into the nuclear genome rather than the genomes of organelles such as mitochondria or chloroplasts. Overall, the *Agrobacterium*-mediated genetic transformation method used in this study effectively introduced foreign genes into the nuclear genome of purslane. And these foreign genes could be stably expressed in purslane cells and transmitted to progeny lines

## 3. Discussion

Purslane is a highly nutritious and versatile plant that holds significant importance in culinary, medicine, and sustainable agriculture fields [2,35,36]. The sequencing and assembly of the high-quality purslane genome have highlighted the importance of studying its gene functions to gain insights into its growth, development, and stress responses [11,37]. Furthermore, transcriptome analysis and single-cell sequencing techniques have been employed to explore gene expression and regulation in *Portulaca oleracea* L., with a particular focus on dissecting the molecular mechanisms involved in the C4/CAM carbon metabolic pathway [10,38]. An increasing number of genes in purslane genome have gained researchers’ attention, and their potential functions have been annotated and analyzed. However, their specific roles in purslane growth and development require further molecular experimental research to be identified. Therefore, the establishment of an effective genetic transformation system is crucial for unraveling the genetic characteristics of purslane and facilitating future advancements in this plant species.

Genetic transformation mediated by *Agrobacterium* largely relies on the regeneration capacity of plant somatic cells [12,26]. Previous studies have revealed that different organ tissues from different plant species exhibit varying abilities of dedifferentiation and redifferentiation [32]. Consequently, the choice of organ tissues used for *Agrobacterium*-mediated genetic transformation varies among different plant species [18]. For rice and maize, an immature embryo is used for callus induction and shoot regeneration. Instead, the leaf is treated as a recipient explant in many plants, including tomatoes, tobacco, etc. [39,40,41,42,43]. The root also exhibits a strong regeneration capacity in many plants, like eggplant, broccoli, and spinach [44,45,46,47]. In this study, different organs of purslane seedlings, including the leaf, stem, root, and seed, were tested for in vitro tissue culture. Among these, leaf explants exhibited the highest callus induction and shoot regeneration rates and were used in the genetic transformation mediated by *Agrobacterium*. Sedaghati et al. also compared the callus induction rate and somatic embryogenesis efficiency of leaf and stem explants [23]. Their results indicated that the stem exhibited a better callus induction response than the leaf but failed to exhibit embryogenic ability. Therefore, for *Portulaca oleracea* L., the leaf might be the appropriate receptor organ for genetic transformation mediated by *Agrobacterium*. Using a leaf explant as the recipient organ for genetic transformation is commonly referred as leaf disc transformation, which was developed by Horsch et al. [40,41]. Currently, leaf disc transformation has become the most widely used method for genetic transformation, taking into consideration that the leaf is easier to sterilize and manipulate in contrast to the stem and root for most plant species.

Numerous previous studies have demonstrated the extensive involvement of hormones, particularly 6-BA and NAA, in regulating various processes such as cell growth, differentiation, and shoot formation during tissue culture [48]. Therefore, these hormones serve as potent tools to manipulate cellular behavior and achieve desired outcomes in both research and practical applications. In the present study, somatic cells from purslane leaf explants exhibited diverse developmental pathways, including callus formation, shoot regeneration, and root differentiation when cultured on media containing different hormone combinations. For instance, the inclusion of 1 mg/L NAA in the growth medium resulted in root differentiation, while the addition of 2 mg/L of the cytokinin 6-BA led to shoot regeneration (Figure 3). Interestingly, when a combination of 2 mg/L 6-BA and 1 mg/L NAA was used, the leaf explants developed into callus tissue instead of roots or shoots. Remarkably, the callus tissue demonstrated the capacity to redifferentiate into shoots upon the removal of NAA from the culture medium (Figure 3). This dynamic interplay between 6-BA and NAA highlights the intricate role of hormones in steering the developmental fate of plant tissue in culture systems.

6-BA is a synthetic cytokinin that promotes cell division and shoot proliferation in plant tissue culture. Moreover, 6-BA facilitates the development of multiple shoots from a single explant, enabling the rapid multiplication of plant material. A series of reports have highlighted the significant regulatory effects of 6-BA on shoot sprouting and shoot proliferation in various plants such as *Bambusa vulgaris* [49], *Bougainvillea buttiana* [50], *Actinidia deliciosa* [51], and *Populus alba* [52]. On the other hand, NAA is a synthetic auxin that plays a key role in root initiation and development in plant tissue culture [53]. By stimulating root growth, NAA promotes the establishment of healthy plantlets with well-developed root systems, which are essential for successful acclimatization [54]. Additionally, NAA can also be used in combination with other hormones to effectively regulate growth and development processes in plant tissue culture [53].

An efficient genetic transformation method depends not only on the plant cell regeneration ability but also on several other factors. The conditions involved in the *Agrobacterium* infection process, including the preculture period, concentration of the *Agrobacterium* suspension, infection and co-culture time, and antibiotic concentration significantly affect the stability and efficiency of the transformation [55,56,57]. Preculture is an important procedure for improving the explants’ survival and transformation rate. Previous studies revealed that genetic transformation efficiency was significantly related to the cell development stage [58,59]. Cells in the active division stage, characterized by active DNA replication, robust metabolism, and a soft and plastic cell wall, are most suitable for genetic transformation [60,61]. In Xia’s research, appropriate preculture significantly increased the number of cells in the division stage and resulted in a higher transformation efficiency in poplar and tobacco leaves. But the appropriate period for different organ tissues from different plant species varied. For most plants, 1–4 days were used for preculture [33,62]. In this study, a 2-day preculture for leaf explants from purslane was executed before *Agrobacterium* treatment. The bacterial concentration, infection duration, and co-cultivation period also affect the transformation efficiency. An excessive bacterial concentration or prolonged infection time and co-culture period can lead to the excessive proliferation of *Agrobacterium*, potentially compromising transformation efficiency. For purslane, previous research showed that the highest transformation frequency was achieved when leaf explants and *Agrobacterium* were co-cultivated for 4 days. Extending the co-cultivation to 6 days did not enhance transformation efficiency and led to bacterial overgrowth and tissue necrosis in the leaf explants [23]. But according to our observations, when the explants were suspended in *Agrobacterium* suspension for 15 min with a bacterial concentration of OD = 0.6, a co-cultivation time exceeding 48 h resulted in uncontrolled *Agrobacterium* growth. Therefore, it is essential to comprehensively consider the *Agrobacterium* suspension concentration, viability, and infection time when determining the co-cultivation duration.

In the genetic transformation method mediated by *Agrobacterium*, two different types of antibiotics are typically used to control *Agrobacterium* growth and screen the transformed cells [6,63]. Timentin, a combination of ticarcillin and clavulanic acid, is widely used in plant genetic transformation due to its ability to eliminate *Agrobacterium* after co-cultivation and its low phytotoxicity toward plant tissues [64]. Kanamycin, hygromycin, and phosphinothricin are commonly employed to select transformed cells, depending on the marker gene on the plasmid carried by *Agrobacterium* [28,39]. To ensure the effectiveness for removing *Agrobacterium* and the efficiency of transformed cell growth and regeneration, the appropriate concentrations used in the process are important. Different plant species exhibit varying responses and tolerances to antibiotics. In our study, 200 mg/L timentin could affectively inhibit *Agrobacterium* growth, and doses up to 400 mg/L did not adversely affect the growth and regeneration of purslane tissues. However, purslane showed a high sensitivity to kanamycin and hygromycin. The results depicted in Figure 4 indicate that a concentration of 25 mg/L kanamycin can stunt seedling growth, and a concentration of 50 mg/L kanamycin can completely inhibit shoot regeneration from explants. Hygromycin was even more inhibitory, with a concentration of 5 mg/L completely preventing the shoot regeneration of purslane explants. Based on our findings, hygromycin is not recommended as the selection antibiotic in purslane genetic transformation. And a concentration of 50 mg/L kanamycin is recommended as the appropriate screening concentration.

## 4. Materials and Methods

### 4.1. Plant Materials and Species Identification

Common wild purslane from Guangzhou, Guangdong Province, China (the accession number in the present study is 0#), was used as the material for plant transformation. The plants were cultivated in the lab for 2 generations to ensure phenotype stability after transplanting from the field. After morphological identification according to the plant appearance, the rDNA sequence was amplified, sequenced using ITS specific primers (ITS-P1: 5′-AGAAGTCGTAACAAGGTTTCCGTAGG-3′, ITS-P4: 5′-TCCTCCGCTTATTGATATGC-3′), and blasted in the NCBI database to further confirm the plant material species.

### 4.2. Sterilization and Aseptic Seedling Preparation

For sterilization, seeds or leaves from soil-planted purslane were first rinsed with aseptic water and 75% alcohol. Then, they were immersed in 84 disinfectant and gently agitated on a shaker for 10 min. Lastly, the seeds or leaves were rinsed 3–4 times with sterile distilled water and placed on sterile filter paper in a petri dish until use. The sterilized purslane seeds were inoculated on MS medium with 30 g/L sucrose and 7 g/L agar and placed in an artificial plant growth chamber (14 h, 26 °C, 3000 lux light/10 h, 25 °C, dark) to germinate and grow.

### 4.3. Callus Induction and Somatic-Cell Regeneration

Different parts of 7-day-old sterile seedlings, including the leaf, stem, and root, were directly cut into appropriate sizes and placed on the medium for callus induction, along with the seeds, to identify the responses of various organs under dark conditions in the growth chamber. Leaves from 2- or 3-week-old soil-planted seedlings also could be used as explants for callus induction and adventitious shoot regeneration. In this study, surface-sterilized leaves from 27 different purslane accessions were cut into 0.5 cm × 0.5 cm pieces and placed on the medium (MS, 30 g/L sucrose, 7 g/L agar, and 2 mg/L 6-BA) to determine their shoot regeneration rate. Additionally, leaf explants of sterile seedlings were inoculated on callus induction medium (MS, 30 g/L sucrose, 7 g/L agar, 2 mg/L 6-BA and 1 mg/L NAA), shoot regeneration medium (MS, 30 g/L sucrose, 7 g/L agar, and 2 mg/L 6-BA), and rooting medium (MS, 30 g/L sucrose, 7 g/L agar, 1 mg/L NAA) at the same time to investigate the development of somatic cells on different media. Subcultures were performed every two weeks. The cultured conditions were as follows: 14 h, 26 °C, 2000 lux light/10 h, 25 °C, dark.

### 4.4. Identification of the Sensitivity to Different Antibiotics

Leaf explants were inoculated on the shoot regeneration medium with 0, 25, 50, 80, 100, 150, or 200 mg/L kanamycin or 0, 50, 10, 25, 50, 80, or 100 mg/L hygromycin for one month, and photos were taken to show the responses of the explants to the antibiotics. For seed explants, seeds were soaked in sterile ddH_2_O with various concentrations of kanamycin or hygromycin for one week. Then, photos of the plants were taken, and the germination rate, root length, and shoot length were measured and analyzed.

### 4.5. Agrobacterium tumefaciens Culture and Plant Transformation

*Agrobacterium* GV3101 with the binary Ti plasmid pCAMBIA1301-35SN was cultured until OD = 0.6–0.8 and then suspended with MS_0_ medium (MS, 30 g/L sucrose, 200 mmol/L acetosyringone) to OD = 0.6. Leaf explants were precultured on the preculture medium (MS, 30 g/L sucrose, 7 g/L agar, 0.2 mg/L 6-BA) for 2 days. The precultured explants were collected, immersed in the *Agrobacterium* suspension for 10 min, and inoculated on the co-culture medium (MS, 30 g/L sucrose, 7 g/L agar, 2 mg/L 6-BA, 200 mmol/L acetosyringone) for 48 h. Then, explants without visible bacteria on their surfaces were selected and inoculated on the shoot regeneration medium supplemented with 200 mg/L timentin instead of acetosyringone for *Agrobacterium* inhibition and shoot regeneration.

For root regeneration, the rootless plantlets were transferred to root induction regeneration medium (MS, 30 g/L sucrose, 7 g/L agar, 1 mg/L NAA) for 2–4 weeks. Then, rooted putative transgenic plantlets were planted in soil for further growth and identification.

### 4.6. GUS Staining and Polymerase Chain Reaction (PCR) Identification

Histochemical assays for GUS activity were conducted in accordance with a previously established protocol outlined by Jefferson et al. (1987), with a few adaptations. Tender leaves were collected and immediately incubated in GUS staining solution containing 1 mM 5-bromo-4-chloro-3-indolyl-β-D-glucuronic acid (X-Gluc: X-Gluc DIRECT, UK), 50 mM sodium phosphate pH 7.0, 1 mM ethylenediaminetetraacetic acid (EDTA), 0.1% Triton X-100, and 10 mM β-mercaptoethanol. Subsequently, the samples were vacuumed for 5 min to facilitate the penetration of the GUS solution into the leaf tissues and then incubated at 37 °C overnight. Then, 70–100% ethyl alcohol was used to decolorize until the color of control plants become nearly transparent.

Total DNA of the regenerated plants and their progeny lines was extracted using a plant genomic DNA isolation kit (B518231, Sangon Biotech (Shanghai, China)) following the manufacturer’s protocol. Polymerase chain reaction using hygromycin specific primers (HYG-F: 5′-CGAGAGCCTGACCTATTGCAT-3′, HYG-R: 5′-CTGCTCCATACAAGCCAACCAC-3′) was used to amplify the foreign gene in the T-DNA strand to verify the transgenic plants. PCR amplification was conducted as follows: 95 °C for 5 min; followed by 30 cycles of 95 °C for 30 s, 56 °C for 30 s, and 72 °C for 30 s; and 72 °C for 5 min. The 481 bp target PCR products were separated by electrophoresis on a 1% agarose gel. A 2000 bp DNA marker was used to indicate the size of target bands. The 2000 bp DNA marker (B50035-500), agarose (A620014-0100), and PCR master mix (B300502-1000) were provided by Sangon Biotech.

### 4.7. Statistical Analysis

Statistical analyses were conducted using IBM SPSS Statistics 23. An analysis of variance was performed, and Duncan’s multiple range test was used to assess the difference between treatments. *p* < 0.05 was considered significant. Significant differences were examined by one-way ANOVA using the LSD test at *p* < 0.05 and *p* < 0.01. The figures were drawn using GraphPad Prism 6.0.

## 5. Conclusions

In this study, the leaf, stem, root, and seed tissues of the wild common *Portulaca oleracea* L. from Guangzhou, China, were utilized as explants for callus induction and shoot regeneration to assess their dedifferentiation and redifferentiation capability. And 27 purslane accessions representing different genotypes were cultured on shoot regeneration medium to identify the efficiency of the medium and the influence of genotype. Based on the optimization of the medium for callus induction and shoot regeneration, as well as the assessment of antibiotic sensitivity, we proposed an effective genetic transformation method mediated by *Agrobacterium* for purslane. GUS staining, specific PCR, and antibiotic selection of the regenerated plants and their progenies demonstrated that the foreign DNA fragment was successfully integrated into the purslane nuclear genome and was stably transmitted to the next generation. Our research has made it feasible to conduct gene function validation within purslane plants through gene overexpression and knockout techniques and enable the possibility of genetic improvement for purslane varieties through precise gene manipulation.

## Figures and Tables

**Table 1:** Origin information of 27 purslane accessions.

Accession No.	City	Province	District	Shoot Regeneration
0#	Guangzhou	Guangdong	South China	Yes
8#	Dehong	Yunnan	Southwest China	Yes
10#	Baise	Guangxi	South China	Yes
17#	Jiulongpo	Chongqing	Southwest China	Yes
21#	Shangqiu	Henan	Central China	Yes
33#	Xi’an	Shanxi	North China	Yes
38#	Guiping	Guangxi	South China	Yes
43#	Jiamusi	Heilongjiang	Northeast China	Yes
46#	Yulin	Guangxi	South China	Yes
47#	Yingtan	Jiangxi	East China	Yes
62#	Zhoukou	Henan	Central China	Yes
64#	Zhenghzhou	Henan	Central China	Yes
65#	Ledong	Hainan	South China	Yes
78#	Datong	Shanxi	North China	Yes
102#	Hechuan	Chongqing	Southwest China	Yes
106#	Zhanjiang	Guangdong	South China	Yes
115#	Suihua	Heilongjiang	Northeast China	Yes
116#	Huainan	Anhui	East China	Yes
117#	Liuyang	Hunan	Central China	Yes
118#	Liuyang	Hunan	Central China	No
119#	Suzhou	Anhui	East China	Yes
320#	Baicheng	Jilin	Northeast China	Yes
326#	Zhoukou	Henan	Central China	Yes
313#	Yuxi	Yunan	Southwest China	Yes
325#	Hefei	Anhui	East China	Yes
500#	Guangzhou	Guangdong	South China	Yes
ZP#	Huai’an	Jiangsu	East China	Yes

## Data Availability

All data are available in the manuscript.

## Supplementary Materials

The following supporting information can be downloaded at https://www.mdpi.com/article/10.3390/plants13172390/s1. Figure S1: Optimization of purslane callus induction medium. Figure S2: Shoot redifferentiation of stem and root explants from purslane. Figure S3: Hygromycin sensitivity identification of seedlings and leaf explants of purslane. Figure S4: Sequence feature information of pCAMBIA1301 vector. Table S1: The concentrations of hormone involved in the medium for shoot regeneration.
