# Supplementary material for: An Effective Somatic-Cell Regeneration and Genetic Transformation Method Mediated by Agrobacterium tumefaciens for Portulaca oleracea L
Source: Plants (Basel). 2024 Aug 27;13(17):2390. doi: 10.3390/plants13172390 (PMC11396874; doi:10.3390/plants13172390)
Supplement: Supplementary file 1 [file plants-13-02390-s001.zip › plants-3092705-supplementary.pdf]

plants

Supplementary Materials

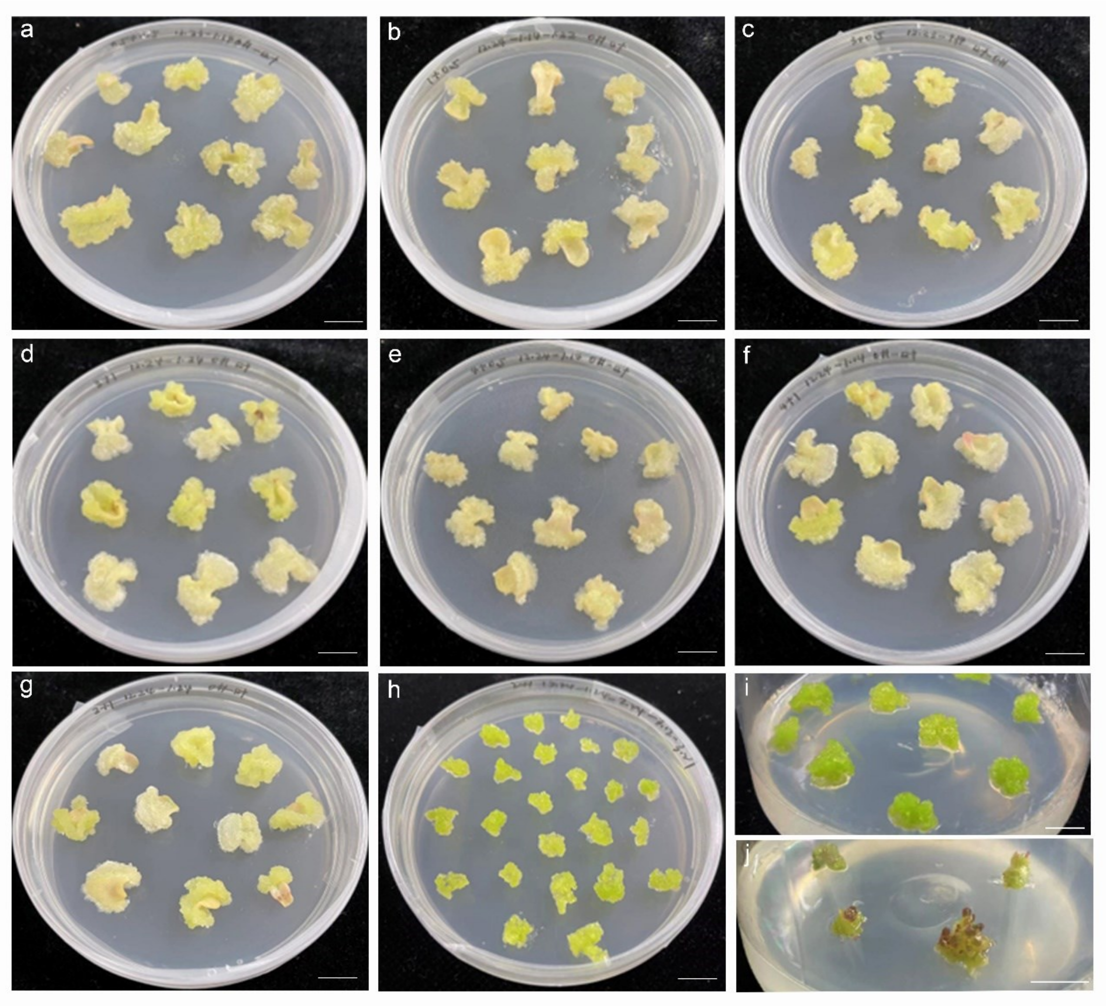

Figure S1. Optimization of purslane callus induction medium. **(a-g)** 30 days callus induction on the basic MS mediums (MS, 30g/L sucrose, 7g/L agar) with various hormone concentrations. **(a)** MS + 0.5mg/L 6-BA + 0.5mg/L NAA. **(b)** MS + 1mg/L 6-BA + 0.5mg/L NAA. **(c)** MS + 3mg/L 6-BA + 0.5mg/L NAA. **(d)** MS + 3mg/L 6-BA + 1mg/L NAA. **(e)** MS + 4mg/L 6-BA + 0.5mg/L NAA. **(f)** MS + 4mg/L 6-BA + 1mg/L NAA. **(g)** MS + 2mg/L 6-BA + 1mg/L NAA. **(h)** Callus after three sub-generations. **(i-j)** Redifferentiation of callus. Bar is 1cm.

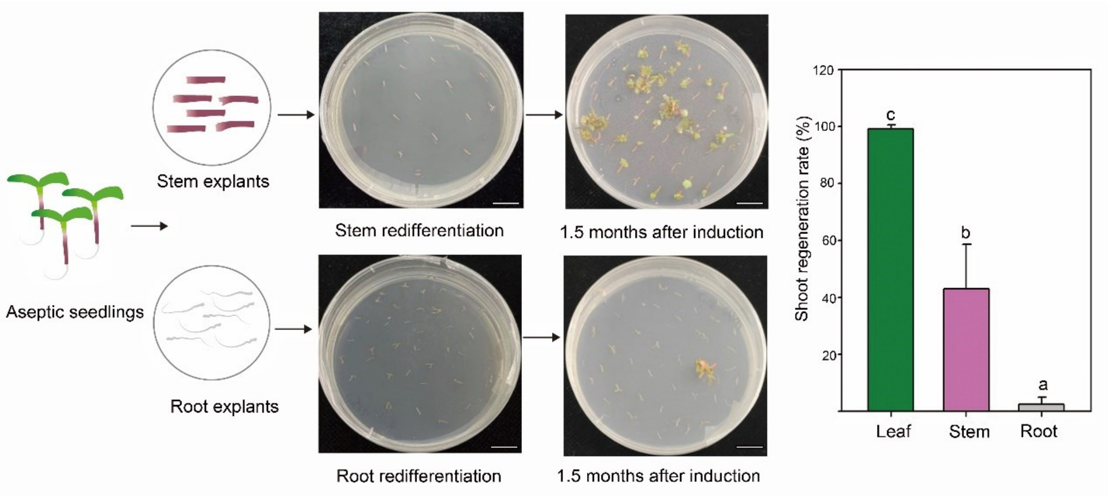

Figure S2. Shoot redifferentiation of stem and root explants from purslane. Bar is 1cm.

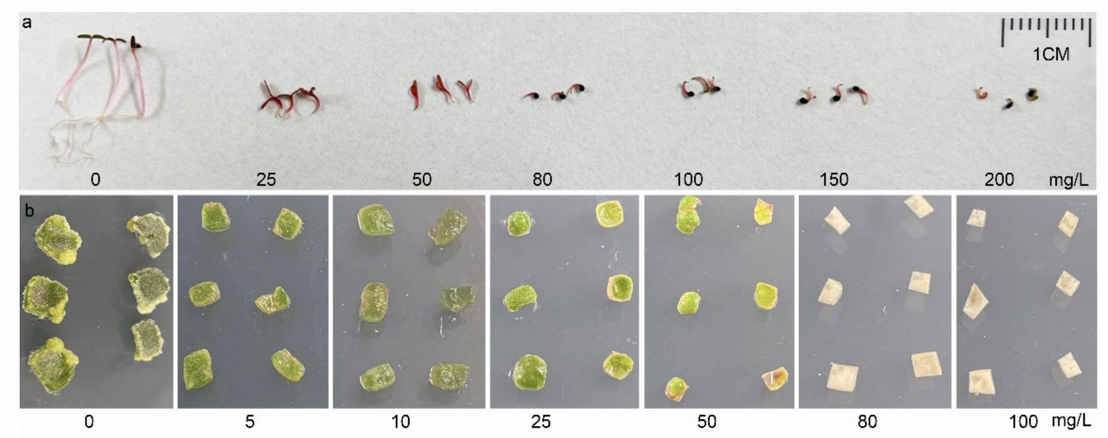

Figure S3. Hygromycin sensitivity identification of seedlings and leaf explants of purslane. **(a)** Purslane seedling growth in the solution with different concentrations of hygromycin. **(b)** Shoot induction of leaf explants on the medium with different concentrations of hygromycin.

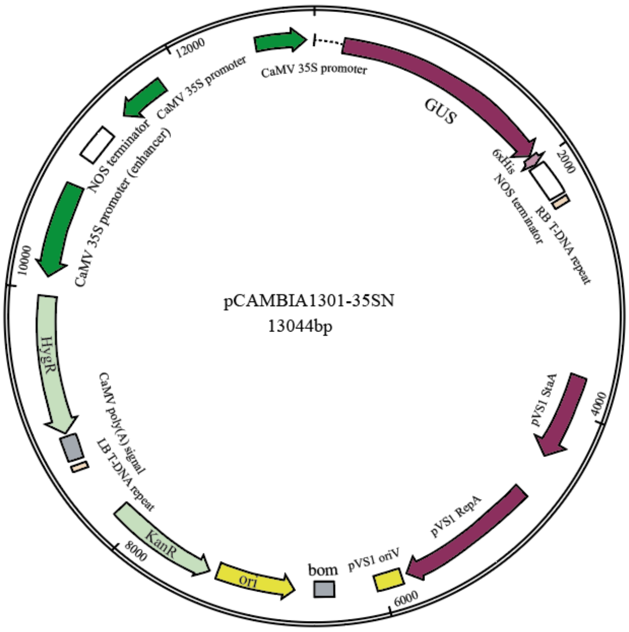

Figure S4. Sequence features information of pCAMBIA1301 vector.

Table S1. The concentrations of hormone involved in the medium for shoot regeneration.

Medium No.	Basic Medium Component	6-BA content	NAA content
1	MS+30g/L sucrose + 7g agar	1 mg/L	0.5 mg/L
2	MS+30g/L sucrose + 7g agar	1 mg/L	1 mg/L
3	MS+30g/L sucrose + 7g agar	1 mg/L	0 mg/L
4	MS+30g/L sucrose + 7g agar	1.5 mg/L	0 mg/L
5	MS+30g/L sucrose + 7g agar	2 mg/L	0 mg/L
6	MS+30g/L sucrose + 7g agar	2 mg/L	0.2 mg/L
7	MS+30g/L sucrose + 7g agar	2 mg/L	0.5 mg/L
8	MS+30g/L sucrose + 7g agar	2 mg/L	1 mg/L
9	MS+30g/L sucrose + 7g agar	2.5 mg/L	0 mg/L
10	MS+30g/L sucrose + 7g agar	3 mg/L	0 mg/L
11	MS+30g/L sucrose + 7g agar	3 mg/L	0.2 mg/L
12	MS+30g/L sucrose + 7g agar	3 mg/L	0.5 mg/L
13	MS+30g/L sucrose + 7g agar	3 mg/L	1 mg/L
14	MS+30g/L sucrose + 7g agar	4 mg/L	0.2 mg/L
15	MS+30g/L sucrose + 7g agar	4 mg/L	0.5 mg/L
16	MS+30g/L sucrose + 7g agar	4 mg/L	1 mg/L
